# Comparative Single-Cell Transcriptomics Reveals Novel Genes Involved in Bivalve Embryonic Shell Formation and Questions Ontogenetic Homology of Molluscan Shell Types

**DOI:** 10.3389/fcell.2022.883755

**Published:** 2022-06-09

**Authors:** David A. Salamanca-Díaz, Elena A. Ritschard, Hannah Schmidbaur, Andreas Wanninger

**Affiliations:** ^1^ Unit for Integrative Zoology, Department of Evolutionary Biology, University of Vienna, Vienna, Austria; ^2^ Division of Molecular Evolution and Development, Department of Neuroscience and Developmental Biology, University of Vienna, Vienna, Austria

**Keywords:** Mollusca, Bivalvia, Dreissena, eco-evodevo, trochophore, larval shell, single-cell seq, proteomics

## Abstract

Mollusks are known for their highly diverse repertoire of body plans that often includes external armor in form of mineralized hardparts. Representatives of the Conchifera, one of the two major lineages that comprises taxa which originated from a uni-shelled ancestor (Monoplacophora, Gastropoda, Cephalopoda, Scaphopoda, Bivalvia), are particularly relevant regarding the evolution of mollusk shells. Previous studies have found that the shell matrix of the adult shell (teleoconch) is rapidly evolving and that the gene set involved in shell formation is highly taxon-specific. However, detailed annotation of genes expressed in tissues involved in the formation of the embryonic shell (protoconch I) or the larval shell (protoconch II) are currently lacking. Here, we analyzed the genetic toolbox involved in embryonic and larval shell formation in the quagga mussel *Dreissena rostriformis* using single cell RNA sequencing. We found significant differences in genes expressed during embryonic and larval shell secretion, calling into question ontogenetic homology of these transitory bivalve shell types. Further ortholog comparisons throughout Metazoa indicates that a common genetic biomineralization toolbox, that was secondarily co-opted into molluscan shell formation, was already present in the last common metazoan ancestor. Genes included are *engrailed*, *carbonic anhydrase*, and *tyrosinase* homologs. However, we found that 25% of the genes expressed in the embryonic shell field of *D. rostriformis* lack an ortholog match with any other metazoan. This indicates that not only adult but also embryonic mollusk shells may be fast-evolving structures. We raise the question as to what degree, and on which taxonomic level, the gene complement involved in conchiferan protoconch formation may be lineage-specific or conserved across taxa.

## Introduction

Mollusca constitutes one of the most diverse metazoan phyla. It is composed of two major subclades, Aculifera and Conchifera, which diverged from one another in the Cambrian (Vinther, 2015; Wanninger and Wollesen, 2015; Parkhaev, 2017; Wanninger and Wollesen, 2019). The Aculifera includes the vermiform, spicule-bearing Solenogastres (Neomeniomorpha) and Caudofoveata (Chaetodermomorpha), as well as the dorso-ventrally flattened Polyplacophora with eight shell plates. The primarily single-shelled Conchifera contains the Monoplacophora, Scaphopoda, Gastropoda, Bivalvia, and Cephalopoda (Kocot et al., 2011; Smith et al., 2011; Vinther, 2015). One molluscan key characteristic is the presence of a mineralized exoskeleton that may come in form of spicules and scales, single or bipartite shells, or serially arranged shell plates. This external armor might have played a crucial role in the evolutionary success of the phylum (Marin et al., 2014).

Molluscan shells and spicules are highly versatile morphological innovations that provide protection and, together with an elaborated musculature, often aid in maintaining structural support (Lowenstam and Weiner, 1989; Simkiss and Wilbur, 2012). They are formed as mineralized secretions from epithelial cells of the mantle (Marin et al., 2007; Furuhashi et al., 2009; Kocot et al., 2016). Once mineralized, shells present a considerable amount of variation in form and shape up to the microstructural level across the different taxa (Chateigner et al., 2000; Furuhashi et al., 2009). In conchiferan mollusks, the shell matrix, i.e., the outer layer of the mantle, is primarily composed of polysaccharides, glycoproteins, chitin, and calcium carbonate (Addadi et al., 2006; Marin et al., 2007). Previous studies that analyzed gene expression in adult mantle tissues of various bivalves and gastropods found that, despite sharing a common set of genes, the expression profiles in the shell matrix differ considerably between taxa, irrespective of their phylogenetic position. This has been used to argue that conchiferan adult shells (teleoconchs) are rapidly evolving features, thus providing an explanation for their high degree of morphological variation across lineages (Jackson et al., 2006; Aguilera et al., 2017; Clark et al., 2020; Yarra et al., 2021).

While conchiferan teleoconchs are continuously secreted from the mantle margin and are highly variable in shape and color, the first-formed embryonic shell (protoconch I) emerges in the gastrula or in the early trochophore larva from the dorsally situated embryonic shell gland (or shell field) in a short time window. It is typically of smooth, non-sculptured appearance (see Wanninger and Wollesen, 2015 for review). Some gastropods with long-lived veliger stages as well as most bivalves form an additional, intermediate shell type, the larval shell (protoconch II) that—similar to its developmental successor, the teleoconch—is secreted from the mantle edge. Only very few studies have focused on the cell lineage, morphological, biochemical, and molecular aspects of the formation of these elusive and microscopic protoconch types (Henry et al., 2004; Kakoi et al., 2008; Lyons et al., 2017; Liu et al., 2018; Liu et al., 2020). While embryonic and larval shell-forming cells have shown to express a common toolbox of markers such as chitin-binding proteins, von Willebrand factor type A domain-containing proteins, and carbonic anhydrases, they display numerous shell matrix proteins (SMPs) that are likely lineage-specific and also differ from those involved in teleoconch formation (Zhao et al., 2018, 2020). However, detailed analyses to assess the number and type of genes that are expressed during protoconch I and protoconch II formation are currently lacking. To fill this gap in knowledge, we reconstructed the shell formation toolbox during protoconch I development in the trochophore larva of the quagga mussel, *Dreissena rostriformis*, using a previously generated single-cell RNA-Seq dataset (Salamanca-Díaz et al., 2022). We also analyzed previously annotated genes which were shown to be expressed in the developing embryonic shell field across conchiferan mollusks for insights into the putative involvement of conserved versus hitherto unknown genes in this key developmental process in the bivalve life cycle.

## Materials and Methods

### Single Cell RNA Sequencing Data Resources

Single cell RNA sequencing data from *Dreissena rostriformis* that had previously been generated (Salamanca-Díaz et al., 2022) were used for the assessment of unknown genes expressed in the shell field as well as for further analyses. In the following, a summary of all major steps from animal acquisition through the in silico analyses performed herein is provided.

### Animal Collection and Cultures

Sexually mature individuals of *Dreissena rostriformis* were collected from the Danube River in Vienna, Austria (N 48°14′45.812″, O 16°23′38.145″). Collection took place between April and September 2019. Adults were gathered from underneath stones and transferred to the laboratory where they were cleaned and maintained in aquaria with filtered river water (FRW) at 19°C.

Spawning of animals was induced by incubating sexually mature specimens in a 10^−3^ M solution of serotonin for 15 min (Sigma-Aldrich, Darmstadt, Germany) in FRW, followed by one wash and subsequent maintenance in FRW. Individuals were kept isolated in FRW in 50 ml glass beakers and after approximately 30 min, up to 50% of the treated specimens started to spawn. Fertilization occurred when three to four drops of sperm-containing water were added to 50 ml glass beakers with oocytes. After fertilization, water was changed every half an hour for the first 3 h and then every 6 h to remove excess sperm and avoid bacterial and fungal growth. The embryos were cultured at 23°C.

### 10X Single-Cell 3′RNAseq

#### Sample Preparation

Cell dissociations of *Dreissena* larvae were generated by first washing 13 h post fertilization (hpf) old trochophore larvae over a 20 µm mesh with sterile media (autoclaved fresh river water; AFRW). Larvae were concentrated and dissociated by first passing them through a syringe with a hypodermic needle with 0.4 mm diameter. A single-cell suspension was loaded into a 10x Chromium Controller using Chromium Single Cell 3’ Kit v2 reagents (Cat #120237, 10xGenomics, United States). cDNA synthesis and library construction were made according to specifications from the manufacturer. Library quantification was performed on a bioanalyzer (High Sensitivity DNA reagents, Agilent Technology #5067-4626; Agilent 2100 Bioanalyzer) and sequenced on the Illumina platform as previously described (Salamanca-Díaz et al., 2022).

#### Mapping Tool Preparation and Cell Clustering

The transcriptomes used for creating the mapping tool and the reference genome used to map the reads against were previously generated (Calcino et al., 2019). In our study, gene models were elongated by 2 kilobases in the 3′ direction to account for poorly annotated three-prime ends in the gene models (Levin et al., 2016). In order to obtain a reference gene nomenclature for the transcriptome of *Dreissena*, we performed a BLASTX search against both human and the Pacific giant oyster (*Crassostrea gigas*) genome for each individual gene sequence. For each transcript, the BLAST hit with the highest E-value was selected for annotation. We utilized InterProScan v5.46-81.0 (Jones et al., 2014) to search for gene ontology and to allocate domains on the reference genome by surveying publicly available databases such as GO terms, Pfam, and PANTHER (Supplementary Table S3). The reference database used in this study was generated by Salamanca-Díaz et al. (2022) using CellRanger Makeref v3.1.0 and demultiplexed using CellRanger Makefastq v3.1.0 with default settings and filtered according to cell barcode and Unique Molecular Markers (UMIs). The resulting cell count gene expression matrix was analyzed in R v3.6.1 (R Development Core Team, 2015) with the Seurat v4.0.1 package (Satija et al., 2015). The count matrix was processed through a standard Seurat pipeline using default parameters. We then generated a KNN graph and clustered the data. Marker genes were identified according to the enrichment and expression of these in at least 10% of the cells in each population (min.pct = 0.1) and with a log fold difference larger than 0.6 (logfc.threshold = 0.6). After this, we selected the differentially expressed genes from the cluster annotated as “shell field” from Salamanca-Díaz et al. (2022) for in-depth homology assessments with respective sequences from other metazoan taxa.

#### Assessment of Unknown Genes and Gene Architecture Annotations

To assess the orthology relationships of shell field-specific genes in the trochophore stage of *D. rostriformis* with genes of other metazoan species, we performed a comparative analysis using OrthoFinder2 (Emms & Kelly, 2019). The genomes, transcriptomes, and gene models for 30 species were analyzed in addition to the previously generated *D. rostriformis* transcriptome and genome assembly (Calcino et al., 2019). These 30 species represent major sub-phylum-level metazoan lineages and were obtained from publicly available data (Supplementary Table S1). At first, proteins were filtered for the longest transcript per gene and used as an input to OrthoFinder. After this, all-versus-all similarity search was obtained using DIAMOND v0.9.15 (Buchfink et al., 2015) and used as input to OrthoFinder2 to identify orthogroups, which are groups of proteins that are likely homologous. Subsequently, proteins belonging to each orthogroup were aligned using MAFFT v7.221 (Katoh & Standley, 2013) for multiple sequence alignments to generate gene trees using FastTree (Price et al., 2009) (-a 16 -b WorkingDirectory -M msa -A mafft -T fasttree). The resulting trees were parsed with the OrthoFinder2 pipeline to discriminate between orthologs and paralogs within each orthogroup. Afterwards, we overlapped these results with the gene sets previously characterized through differentially expressed genes in the single-cell RNA sequencing of the shell field. This resulted in identification of the orthogroups which contain differentially expressed genes in the shell field of the trochophore larva.

**FIGURE 1:**
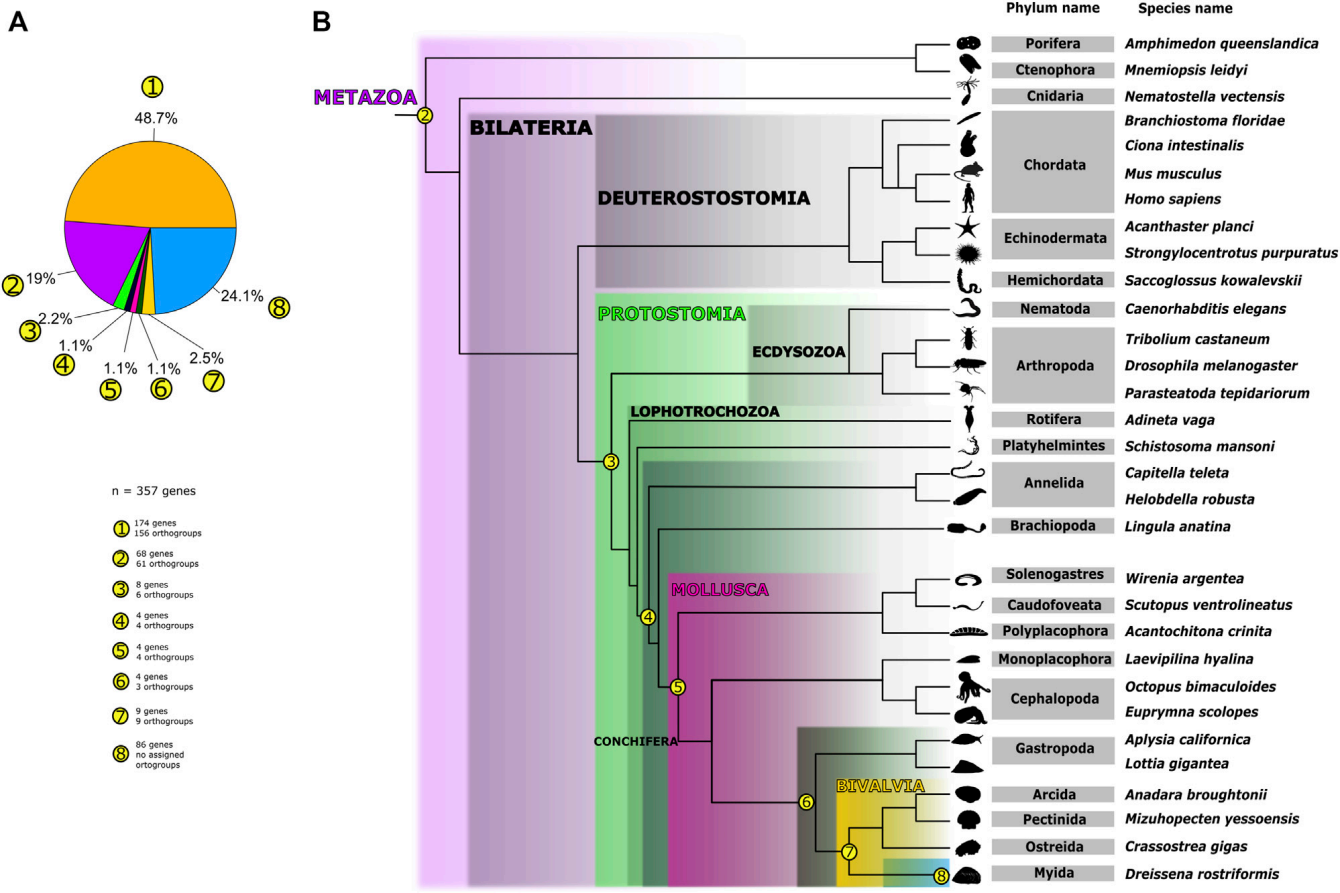
Distribution of orthogroups containing shell field-specific genes from *Dreissena rostriformis* across Metazoa. **(A)** Pie chart representing the number of orthogroups and genes found in the respective taxa. Each subset of orthogroups is numbered (1-8), indicating how many shell field-specific genes are contained in each taxon, together with the total amount of shell field-specific genes analyzed. **(B)** Dendrogram representing phylogenetic relationships of the sampled species and the presence of orthogroups and genes on each node. Phylogenetic relationships of the sampled species are plotted on a class-level tree based on previous studies (Smith et al., 2011; Laumer et al., 2019; Lemer et al., 2019; Fernández and Gabaldón, 2020; Li et al., 2021). Numbers correspond to those in **(A)**. Number 1 refers to all shell field orthogroups that are randomly distributed (i.e., diverse and without distinct pattern) among the sampled metazoans (48.7%). Numbers 2–8 depict the shell field genes/orthogroups identified for the respective nodes in the phylogeny. Node (2) refers to the 19% of all shell field orthogroups present in all sampled metazoan genomes in this study. Node (3) is equivalent to 2.2% of all shell field orthogroups present in sampled protostome organisms. Node (4) corresponds to the 1.1% of orthogroups present in the sampled organisms classified as Lophotrochozoa. Node (5) refers to all shell field orthogroups present exclusively in the sampled mollusks (1.1%). Node (6) represents all shell field orthogroups (1.1%) present in the gastropod and bivalve genomes sampled. Node (7) depicts all shell field orthogroups (2.5%) present in bivalve genomes analyzed here. Node (8) represents all *D. rostriformis*-specific shell field genes that could not be assigned to any orthogroup. Species silhouettes were obtained from www.phylopic.org and are either licensed under Creative Commons Attribution 3.0 Unported or are available under public domain.

For insights into the architecture of genes that are differentially expressed in the shell field, we used the webserver of SignalP v5.0 with default parameters (Almagro Armenteros et al., 2019) to search for signal peptides in each corresponding sequence. Additionally, TMHMM v2.0 webserver (Krogh et al., 2001) was used to screen transmembrane domains and predict which amino acid sequences have domains on the outer side of the plasma membrane. For insights into the tertiary structure of the peptide sequence of each gene from this set, we used the Phyre2 webserver (Kelley et al., 2015). Further gene annotations, corresponding to Pfam, PANTHER, GO term, human, and *Crassostrea gigas* ortholog similarity, were implemented from a previous study (Salamanca et al., 2022). Gene expression levels of the 17 existing transcriptome libraries (Calcino et al., 2019) were quantified with Kallisto (transcripts per million, TPM) (Bray et al., 2016). Expression data from *Crassostrea gigas* were collected from public databases (Supplementary Table S1) and TPM values were calculated following the pipeline of a previous study (Zieger et al., 2021). Heatmaps showing normalized quantitative expression of genes were plotted with R (R Developement Core Team, 2015) with the heatmap function from the ComplexHeatmap R package (Gu et al., 2016) (Figure 1, Supplementary Table S3).

## Results

### Overall Orthogroup Statistics of the *Dreissena rostriformis* Genome

To discard false positives while screening for novel genes in the shell field, the orthology assessment was made using the whole genome of *Dreissena rostriformis*. After that, we analyzed the genes that are exclusively part of the transcriptomic signature from the shell field of the trochophore larva. Around one third of all orthogroups (31.4%) predicted from 30 different metazoan species contain *Dreissena rostriformis* (“DRERO”) genes (cf. Supplementary Table S3). In addition, we identified *D. rostriformis* lineage-specific orthogroups with non-annotated genes, meaning there is a noteworthy number of genes that have no known match with any other animal sampled. However, all other species used in our analysis show similar low percentages of genes that can be assigned to known orthogroups (Supplementary Table S3), corroborating the common notion of the vital role of lineage specific genes or families during animal genome evolution (Fernández and Gabaldón, 2020). In *D. rostriformis*, such genes identified from the genome mount up to 19.8% (7469 genes; see Supplementary Tables S2, S3).

### Orthogroups Containing Genes From the Trochophore Shell Field

**FIGURE 2:**
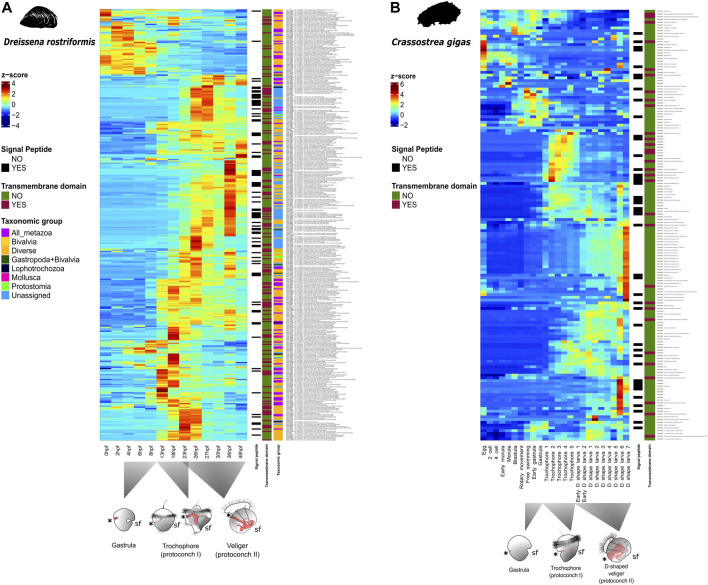
Relative quantitative expression of shell field-specific genes during development of two bivalve species. **(A)** Heat map showing relative normalized expression levels for each isolated gene from the shell field of the trochophore of *Dreissena rostriformis*. Normalized gene expression (transcripts per million; TPM) is depicted in graded shades of red when values are above the median, those below this threshold and with a value close to zero are in shades of blue. Details on gene annotations, orthogroup assignments to the respective taxonomic level, presence of signaling peptides, and transmembrane domains are provided in Supplementary Table S2. Blast top hit to the Pacific oyster is next to each gene name. Time after fertilization (hpf) and corresponding developmental stages at 23°C are ordered chronologically at the bottom of the *x* axis. Germ layers and their major derivatives in animal schemes are depicted in grey (mesoderm), red (endoderm), and white (ectoderm), respectively. Asterisks mark the blastopore/mouth, sf indicates the shell field. **(B)** Heat map showing relative normalized expression levels of genes isolated from larval and adult shells of *Crassostrea gigas* as described in Zhao et al. (2018). Normalized gene expression is depicted in graded shades of red when values are above the median, those below this threshold and with a value close to zero are in shades of blue. Each developmental stage is organized chronologically from left to right on the *x* axis. Details on gene annotations, orthogroup assignments, presence of signaling peptides, and transmembrane domains are provided in Supplementary Table S9 (cf. Zhao et al., 2018).

Using the outputs from the OrthoFinder and Single-cell seq pipelines, we characterized the shell field-specific genes and their orthogroup correspondence (Supplementary Table S2). In total, we analyzed 357 genes differentially expressed in shell field cells from the trochophore stage of *Dreissena rostriformis*. Gene ontology terms of these genes showed enrichment in shell formation-associated processes such as vesicle-mediated transport, phospholipid metabolic processing, integrin-mediated signaling pathway, and positive regulation of cell cycle G2/M phase progress (Salamanca-Díaz et al., 2022; Supplementary Table S2). Tertiary structure analysis using the Phyre2 webserver coincide with and thus confirm the results from SignalP and Interproscan (Supplementary Tables S2, S5, S6 and Supplementary File S1). In addition, expression dynamics of shell matrix genes during development of the trochophore of *D. rostriformis* were analyzed and compared with shell field-specific genes from pre-metamorphosis stages of the oyster *Crassostrea gigas* using previously published RNA-seq data (Figure 2, Supplementary Tables S7, S8) (Zhao et al., 2018; Calcino et al., 2019). Expression of most of these genes starts early in development, i.e., shortly after fertilization, likely by maternal transcripts. High normalized peaks of transcription are seen throughout the late gastrula and trochophore stages (during which the protoconch I is established) and continue in the veliger stages (continuous protoconch II formation) *i.e.*, between 13 and 48 hpf. *In situ* hybridization experiments of some of these genes have previously shown a high level of expression in stages of embryonic shell (protoconch I) formation (e.g., *Hox 1*, *hic31*) (Salamanca-Díaz et al., 2021; Salamanca-Díaz et al., 2022). Furthermore, numerous orthogroups that contain genes that are specific to the embryonic shell field are shared across Metazoa (Supplementary Tables S2, S4. This demonstrates multiple cooption events of these genes into various functions in the respective metazoan lineages (Supplementary Table S1). However, we also found a group of genes (68 genes in 61 orthogroups; e.g., *engrailed*, *cyclin-A2, carbonic anhydrase*, *tyrosinase* homologs) active in *D. rostriformis* shell field formation that are also involved in shell formation of other mollusks (Nederbragt et al., 2002; Iijima et al., 2008; Kin et al., 2009; Samadi and Steiner, 2009; Huan et al., 2020; Zhao et al., 2020). These genes are also present in all other metazoans screened for herein and are commonly known to be related to body plan specification, cell cycle, and metalloenzyme activity (Figure 1 and Supplementary Table S2).

A closer analysis of specific taxonomic orthogroups (e.g., Protostomia, Lophotrochozoa) revealed that the majority of the genes (48.7%) that are differentially expressed in the shell field in *D. rostriformis* have orthologs in other taxa. However, their distribution between the given taxa is highly variable (Figure 1, Supplementary Tables S2, S4). While 19% of the shell field-specific genes are shared with other metazoan taxa, only 2.24% of the total number of shell field-specific genes are shared with other protostome species (8 genes in 6 orthogroups), and 1.1% of the same total number of genes are restricted to lophotrochozoans (4 genes in 4 orthogroups). Within molluscs, 1.1% of the shell field-specific genes are shared with other conchiferans, another 1.1% were only found in the sampled bivalves and gastropods, and 2.5% are possibly bivalve-specific. A quarter of the shell field-specific genes were only found in *D. rostriformis* and are not shared with other taxa. These may either genus- or species-specific genes, however their evolutionary history needs to be assessed in more depth once more bivalve datasets become available. The majority of genes in all orthogroups do not have a match in the InterPro database but show low level similarities with human orthologs (e-values higher than 1). Among the few genes with annotations in these groups, there is a member of the Claudin protein family, a *keratin* ortholog, epidermal growth factor domains, *heat shock 70 kDa protein*, and an *endonuclease 2* ortholog. Genes expressed in the shell field which are restricted to Mollusca lack confident annotations (Supplementary Table S2). Human blast hits show only few domain commonalities to genes with a role in protein modification, DNA repair, nuclear envelope component and ion exchange, i.e., *DDB1 and CUL4 associated factor 4*, *DNA repair protein XRCC1, nuclear envelope integral membrane protein 2*, and *sodium-driven bicarbonate exchanger*.

The number of hitherto non-annotated genes shows a tendency to decrease when analyzing the different lineages inside Mollusca. This suddenly changes in the branch leading to *Dreissena rostriformis*, where the number of shell field-specific genes notably increases (Figure 1). Within Conchifera, a putative Bivalvia + Gastropoda clade shows 2 hitherto undescribed genes in 2 separate orthogroups and Bivalvia alone presents a unique set of 9 genes in 9 orthogroups (Figure 1, Supplementary Table S2). In this gene set, there are low e-values and little similarity to human as well as *Crassostrea gigas* orthologs, with blast hits to genes associated to antioxidant reactions, cell migration, cell attachment, and cellular proliferation, i.e., *superoxide dismutase*, *myomegalin*, *laminin subunit beta-4*, and *ETS domain-containing transcription factor ERF*. Additionally, from the genes expressed in the *D. rostriformis* embryonic shell field which were not assigned to any orthogroup or have a specific identity, and thus are considered here for *Dreissena* to be lineage*-*specific (86 genes in total, 24% of all shell field genes), 39 have transmembrane domains and 41 have signal peptides. This suggests that almost half of this gene subset is probably crucial for cell signaling since it has domains that interact directly with the outside of the cell membrane (Supplementary Tables S2, S5, S6). Altogether, our results show that, while there is a core gene set expressed in the embryonic shell field which is present throughout Metazoa, there is also strong indication of novel gene emergence that is specific to the embryonic shell field of the *Dreissena* trochophore.

## Discussion

### High Number of Putative Novel Lineage-Specific Genes Involved in Embryonic Shell Formation

Previous studies have characterized the shell secretomes from larval and adult stages of two marine bivalves, *Pinctada fucata* and *Crassostrea gigas*. They found that, despite having some common gene expression signatures (e.g., *carbonic anhydrase*, *chitin binding protein*, and *von Willebrand factor type A*), they also show distinct expression patterns of larval shell matrix proteins depending on species and developmental stages. One significant subset of the genes (around 90 out of 156 genes) involved in shell secretion is expressed in trochophore stages, while the other genes are expressed during the later D-shape veliger stages, suggesting different molecular signatures underlying embryonic versus larval shell formation (Zhao et al., 2018). This calls into question the homology of embryonic and larval shells in Bivalvia. Since solid data on the genes involved in bivalve teleoconch formation are still lacking, evolutionary relationships between the adult and the two transitory protoconch shell types currently remain unknown. This underlines that more in-depth comparative studies are needed to assess the decades-old question of (ontogenetic) homology of conchiferan embryonic, larval, and adult shells within the respective sublineages (particularly bivalves, gastropods, and scaphopods).

Our study shows that 24% (86) of the genes differentially expressed in the shell field of the trochophore of *D. rostriformis* could not be assigned to any orthogroup and may thus be genus- or species-specific (Figure 1A). From these, 13 unassigned genes have only incomplete annotations in specific regions of each gene sequence, 41 have low e-value similarity with human or *Crassostrea* orthologs, and 32 of these genes have no known annotation or ortholog match (Supplementary Table S2). Similar trends are also known from other mollusks, where unassigned and undescribed genes expressed in shell- and plate-forming cells appear to be highly taxon-specific. For example, secretomes from adult gastropods, bivalves, polyplacophorans, and a nautiloid cephalopod show considerable levels of lineage-specific orphan genes (Jackson et al., 2006, 2009; Immel et al., 2016; Kocot et al., 2016; Marin, 2020; Setiamarga et al., 2021). It has been argued previously that the rapid evolutionary rate of these genes may be a possible reason for the lack of orthology detection of these shell matrix toolbox genes (Aguilera et al., 2017). This, in turn, could be the result of evolutionary responses to the widely varying ecological conditions shell-bearing mollusks are exposed to, since most of the gene products in the shell field are in direct contact with the environment. Interestingly, almost half of these lineage-specific orphan genes have transmembrane domains and/or signaling peptides (Supplementary Tables S2, S5, S6). This suggests that genes expressed in the shell field at the trochophore stage might be significantly influenced by the ecology of the larva. Previous studies have found that molluscan shell proteomes drastically change when ecological factors such as the pH or the temperature are altered, but combined experimental and transcriptomic studies are currently too scarce for robust conclusions on an evolutionary level (Timmins-Schiffman et al., 2014; Wei et al., 2015). However, since the environmental conditions during protoconch I and protoconch II formation are identical in *D. rostriformis*, this might hint towards an independent evolutionary origin (and thus argue against ontogenetic homology) of these shell types. This is further supported by the fact that, after shell field formation, there is a fluctuation of gene expression throughout development, *i.e.*, *Chitin binding domain* ortholog (Gene.49769) and *voltage-dependent calcium channel subunit alpha-2/delta-4* human ortholog (Gene.25093) (Figure 2A), demonstrating putatively different expression dynamics during protoconch I and protoconch II formation, respectively. A similar tendency emerges when comparing temporal expression dynamics of shell-specific genes of *D. rostriformis* with *C. gigas* (Figure 2B). The Pacific oyster seems to have different sets of genes with alternate expression throughout developmental stages where shell field formation is active, just as in *Dreissena,* thus calling into question the homology of bivalve ontogenetic shell types (cf. Zhao et al., 2018). However, further comparative studies employing different developmental stages of the same as well as similar developmental stages of different species are needed to further assess this assumption.

### Metazoan Biomineralization Gene Repertoires

Our single-cell RNAseq and OrthoFinder analyses grouped 271 (76%) out of 357 identified genes that are differentially expressed in shell field cells from the trochophore stage of *Dreissena rostriformis* into orthogroups shared with different taxa. From these resulting orthogroups, there is a fraction of *Dreissena* trochophore shell field-specific genes that are shared with the rest of the sampled metazoans (68 genes in 61 orthogroups, 19%) (Figure 1, Supplementary Table S2). It has been shown previously that mantle secretomes in other bivalves and gastropods also possess a wide range of gene families that originated prior to the emergence of the conchiferan clade (Kocot et al., 2016; Aguilera et al., 2017). Among this, a set of genes from the shell field of *Dreissena*, which are present in other metazoans, is known to be involved in extracellular matrix formation, such as orthologs of *laminin*, *C-type lectin* domains, and immunoglobulins (Supplementary Table S2). Additionally, among this set there are genes containing leucine-rich repeat domains and semaphorins, which are also found throughout metazoans (Supplementary Table S2). Moreover, genes from this subset of orthogroups were coopted into embryonic shell formation in conchiferan mollusks such as *Dreissena*, e.g., *Hox 1* (Gene.152834)*, Hox 4* (Gene.66474)*, Lox 4* (Gene.142102)*,* and *engrailed* (Gene.126286) (Figure 1, Supplementary Table S2) as previously described for other species of mollusks (Samadi & Steiner, 2009; Fritsch et al., 2015; Wollesen et al., 2018; Huan et al., 2020; Liu et al., 2020; Salamanca-Díaz et al., 2021). Furthermore, in these orthogroups there are genes that have been found to be also involved in biomineralization processes in echinoderms and vertebrates, e.g., *Cyclophilin-type* (Gene.103270) and *Carbonic anhydrase* (Gene.82229) (Livingston et al., 2006; Mann et al., 2008; Mann et al., 2010; Mann and Edsinger, 2014). Such an organic matrix is formed prior to secretion of the mineralized part of the shell and is thus of crucial importance for conchiferan mollusks, but the respective factors involved are also present in other metazoans that lack a shell (Supplementary Table S2) (Marie et al., 2010; Marie et al., 2011a; Marie et al., 2011b; Marie et al., 2012; Marin et al., 2014). Altogether, our data point towards a shared “molecular biomineralization toolbox” across Metazoa, but a broader taxon sampling especially from key invertebrate phyla are required for deeper evolutionary insights. Given the fact that numerous animal phyla contain taxa with mineralized hard parts, including accessible representatives such as annelids, brachiopods, other lophotrochozoans, as well as numerous arthropods, this hypothesis can be tested by comparative studies using single-cell RNA transcriptomic approaches.

Taken together, the quagga mussel *Dreissena rostriformis* shows a mosaic of co-option of known metazoan genes and *de novo* recruitment of genes with hitherto unknown function or ortholog match into embryonic (protoconch I) shell formation. Our data suggest that not only adult but also embryonic bivalve shells are highly plastic in the gene repertoire that underlie their ontogeny, which may be indicative of non-homology of bivalve—and possibly conchiferan - ontogenetic shell types.

## Data Availability

The data presented in this study are deposited in NCBI’s Gene Expression Omnibus and are accessible through the GEO series accession number GSE192624 (https://www.ncbi.nlm.nih.gov/ geo/query/acc.cgi?acc=GSE192624).

## Supplementary Material

Supplementary Table S1 Genome and proteomes of publicly available data. Columns show species scientific names, common names, their respective abbreviation used in this study, databases where the data were obtained from, as well as the molecular nature (either genome or transcriptome assembly) of each sample.

Supplementary Table S2 Annotations of shell field genes from *Dreissena rostriformis*. Results show the orthofinder, InterProScan, TMHMM, SignalP, and BLAST searches against the human genome. Each column corresponds to the gene code from the quagga mussel, the analysis performed on the aminoacid sequence, the signature accession result of the analysis, signature description, accession code on the InterPro database, InterPro description, associated gene ontology terms, similarity index of the analysis (e-value), blast top hit against the human genome and the Pacific oyster with the respective e-value, presence of transmembrane domains and signal peptides, orthogroup containing the corresponding gene and the taxonomic clades containing that orthogroup, and the length of the protein sequence of each gene.

Supplementary Table S3 Statistics result of the orthofinder analysis showing the number of genes assigned to each orthogroup for all species used in the analysis.

Supplementary Table S4 Resulting orthogroups that contain at least one shell field gene obtained from the single cell RNA seq analysis from *D. rostriformis*.

Supplementary Table S5 Transmembrane helices domains predicted for trochophore shell field genes from *D. rostriformis* with the TMHMM 2.0 webserver.

Supplementary Table S6 Signal peptide-positive proteins prediction for the trochophore shell field genes from *D. rostriformis* with the SignalP 5.0 software.

Supplementary Table S7 Raw data of relative expression levels (transcripts per million, TPM) of *Dreissena rostriformis* shell field genes throughout development.

Supplementary Table S8 Raw data of relative expression levels (transcripts per million, TPM) of *Crassostrea gigas* shell field genes throughout development [based on Zhao et al. (2018)].

Supplementary Table S9 Annotations of shell-related genes from *Crassostrea gigas.* Results from annotations of shell protein matrix genes taken from Zhao et al. (2018). Each column corresponds to annotations assigned in Zhao et al. (2018). Indicated is the gene code from the NCBI database, the result of the analysis performed on the aminoacid sequence showing the presence of transmembrane domains and signal peptides, and the assigned signature description.

Supplementary File S1 Output generated from the Phyre2 webserver. Summary results and files are in pdb format for the predicted tertiary structure of each amino acid sequence of the dataset.
